# Machine learning driven non-invasive approach of water content estimation in living plant leaves using terahertz waves

**DOI:** 10.1186/s13007-019-0522-9

**Published:** 2019-11-18

**Authors:** Adnan Zahid, Hasan T. Abbas, Aifeng Ren, Ahmed Zoha, Hadi Heidari, Syed A. Shah, Muhammad A. Imran, Akram Alomainy, Qammer H. Abbasi

**Affiliations:** 1 James Watt School of Engineering, University of Glasgow, Glasgow, UK; 2 School of Electronic Engineering, Xidian University, Xi’an, Shaanxi China; 3 School of Electronic Engineering and Computer Science, Queen Mary University of London, London, UK

**Keywords:** Water content, Plant leaves, Terahertz (THz), Sensing, Agriculture, Classification, Machine learning

## Abstract

**Background:**

The demand for effective use of water resources has increased because of ongoing global climate transformations in the agriculture science sector. Cost-effective and timely distributions of the appropriate amount of water are vital not only to maintain a healthy status of plants leaves but to drive the productivity of the crops and achieve economic benefits. In this regard, employing a terahertz (THz) technology can be more reliable and progressive technique due to its distinctive features. This paper presents a novel, and non-invasive machine learning (ML) driven approach using terahertz waves with a swissto12 material characterization kit (MCK) in the frequency range of 0.75 to 1.1 THz in real-life digital agriculture interventions, aiming to develop a feasible and viable technique for the precise estimation of water content (WC) in plants leaves for 4 days. For this purpose, using measurements observations data, multi-domain features are extracted from frequency, time, time–frequency domains to incorporate three different machine learning algorithms such as support vector machine (SVM), K-nearest neighbour (KNN) and decision-tree (D-Tree).

**Results:**

The results demonstrated SVM outperformed other classifiers using tenfold and leave-one-observations-out cross-validation for different days classification with an overall accuracy of 98.8%, 97.15%, and 96.82% for Coffee, pea shoot, and baby spinach leaves respectively. In addition, using SFS technique, coffee leaf showed a significant improvement of 15%, 11.9%, 6.5% in computational time for SVM, KNN and D-tree. For pea-shoot, 21.28%, 10.01%, and 8.53% of improvement was noticed in operating time for SVM, KNN and D-Tree classifiers, respectively. Lastly, baby spinach leaf exhibited a further improvement of 21.28% in SVM, 10.01% in KNN, and 8.53% in D-tree in overall operating time for classifiers. These improvements in classifiers produced significant advancements in classification accuracy, indicating a more precise quantification of WC in leaves.

**Conclusion:**

Thus, the proposed method incorporating ML using terahertz waves can be beneficial for precise estimation of WC in leaves and can provide prolific recommendations and insights for growers to take proactive actions in relations to plants health monitoring.

## Background

The growing consciousness of fruits and vegetable quality in recent years, while utilizing natural resources such as water consumption [1], strongly demand viable and feasible techniques to detect early symptoms of plants drought stresses [1, 2]. The recent climate transformations and growing deficiency of water resources have posed enormous challenges, particularly in the applied plant biology sector [3, 4]. In this regard, much efforts have been geared by researchers, horticulturists, and plant physiologists at various levels in the plant science sector, towards developing feasible strategies for non-invasive techniques [5–9] in monitoring the health status, and biological traits of leaves to sustain crops productivity. Hence, a precise estimation of water content (WC) at a cellular level in plants leaves is of high-importance to growers, and cultivators to take appropriate and efficient measures by facilitating them with appropriate amounts of resources inputs, i.e. water and nutrients to maintain healthy physiology [3–9].

In recent years, many conventional techniques [6–13] have been suggested for accurate estimation of WC in leaves and studied the morphological structure of leaves in detail. These methods including magnetic resonance imaging (MRI), near-infrared spectroscopy (NIRS), hyper-spectral imaging [8–13] have offered better reliability but have been suffered by some limitations and considered as time-consuming, and unsuitable for long-term studies due to disparaging nature [9–13]. Besides, some others non-destructive techniques such as thermal imaging [12–16] have been proposed, and yet they too are littered with limited resolution and sensitivity issues, and transpired as inappropriate for detecting monitoring information on water dynamics and diminutive changes at the cellular level [13–16]. Consequently, the evolving applications of terahertz time-domain spectroscopy (THz-TDS) technology, which is considered as non-intrusive, has been deployed in the field of plant physiology to detect anomalies proactively and investigate the structural behaviour and complex traits of leaves under the particular environment [16–18]. This technique is proven to be more effective and reliable compared to other approaches. However, it is a costly technique, and on-site access is limited [16–18].

Meanwhile, terahertz (THz) technology has been widely used in diverse field applications such as diagnostic applications of dental and skin-care [4, 19, 20], unseen hazard items [5], material characterizations [4, 5], and telecommunications [5, 20]. However, researchers from plants science sector are of the strong view that its potential to disseminate through plants sector is still to be thoroughly revealed, considering it as a new source of vital improvements for the agricultural sector [4, 21]. The aforesaid prevailing challenges in exploring the spectral analysis of WC in leaves using THz have immensely engaged numerous scientists and captivated researchers from diverse fields. Moreover, evidence from multi-disciplinary agri-technology studies show that reliable and early detection of WC in plants leaves at a cellular level can drive agricultural productivity and optimize the economic benefits [10–12]. For this purpose, machine learning (ML) applications create an innovative opportunity to unravel, quantify, and understand data-intensive processes in agricultural operational environments [22]. In recent time, the applications of ML have been immensely used in various scientific fields [22] such as healthcare sector, food security, meteorology, medicine, meteorology, economic sciences [22]. Furthermore, researchers are very keen to discover its possibilities, specifically in modern digital agriculture systems to develop intelligent management of plants by applying the water distribution effectively [22].

Considering the sensory characteristics of plants leaves, water is essential to the overall growth, transpiration, and nutritional process of plants leaves [10]. Therefore, timely delivery of the appropriate amount of resource inputs such as water and its precise quantification can be very beneficial to drive and sustain overall crops productivity in an advanced agricultural system [10]. This paper presents a state-of-the-art method to closely monitoring the water dynamics in leaves using the scattering parameters of THz pulse waves through ML. In our study, we demonstrated that there is a clear relationship between the parameters of the pulse wave and the plants WC within a frequency range from 0.75 to 1.1 THz. We have performed in-lab experiments using three different plant leaves, including coffee, pea-shoot, and spinach for four consecutive days. Subsequently, the data is pre-processed for feature extraction and is fed to our proposed ML algorithm for automated classification of WC on different days.

The overarching aim of this study is to estimate and predict the future trends of WC in plants’ leaves in an automated fashion using THz pulse waves, which is indicative of the health status of the plants. For this purpose, we have extracted time and frequency domain-features of THz pulse wave and use it to train ML models to monitor WC in coffee, pea-shoot and spinach more precisely. By performing the leave-one-observation-out cross-validation, we strongly feel that our proposed model has the capability to monitor the WC future trend proactively. Hence, it can save crops from stresses by taking timely action, which will ultimately help to increase yield production and optimize economic benefits. The rest of the paper is structured as follows: “Methods” presents methods and the implemented methodology for data collection and pre-processing, along with an initial classification accuracy of primary data. This is followed by the description of the feature extraction technique in “Results”. Section VI describes the proposed classification algorithms and optimal parameter selection method. In “Conclusion” and VI, the feature section and analysis of three classifiers results are discussed, respectively. Finally, the conclusion is drawn out in section VI.

## Methods

### Experimental setup

**Fig. 1:**
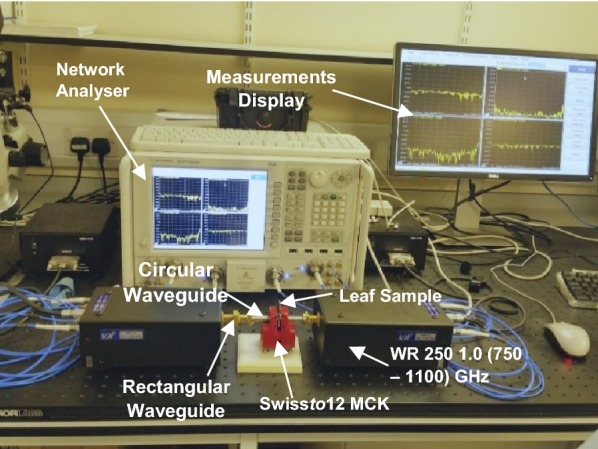
Experimental setup of Swiss*to*12 MCK system used for measurements of leaves in the frequency range from 0.75 to 1.1 THz

In this setup, a THz Swiss*to*12 Material Characterization Kit (MCK) [23] was employed to obtain the scattering parameters of three plant leaves. The MCK was connected to a Virginia Diodes Analyzer (VNA) extender WM-250 (WR1.0) which operated in the frequency range of 0.75 THz to 1.1 THz. The structural integrity and configuration of leaves were also considered by employing two Polytetrafluoroethylene (PTFE) caps which were fitted internally to the waveguide and could provide a consistent compression to samples, as shown in Fig. 1. Prior to any measurements, the setup was calibrated using the two-port short-open-load-thru (SOLT) calibration technique to confiscate any unwanted errors or noicse that may have occurred while performing measurements.

### Sample

Three various kinds of plants leaves were used for measurements are coffee-arabica, pea-shoot and baby-spinach. In this study, these fresh leaves were detached from plants, which were fully grown and nurtured in Rouken Glen Farm, East Renfrewshire, Glasgow. According to the status of these plants, these leaves grew well with no pests or disease and were kept in the laboratory under the environment temperature of 18 °C ± 0.1 °C, and the humidity was between 20% ± 2%. The thickness and weight of the leaves were continuously monitored for four consecutive days using the Vernier calliper and electronic scale, respectively. The thickness of leaves appeared to decrease substantially due to leaf dehydration. Hence, variations in WC of leaves was the key factor that caused spectral variation in measurements, as shown in Fig. 1. In addition, all leaves’ thickness and weight were measured at three various locations after every 120 min during the natural evaporation of WC to analyse the unevenness in the surface of leaves.

### Procedure for data collection and pre-processing

**Fig. 2:**
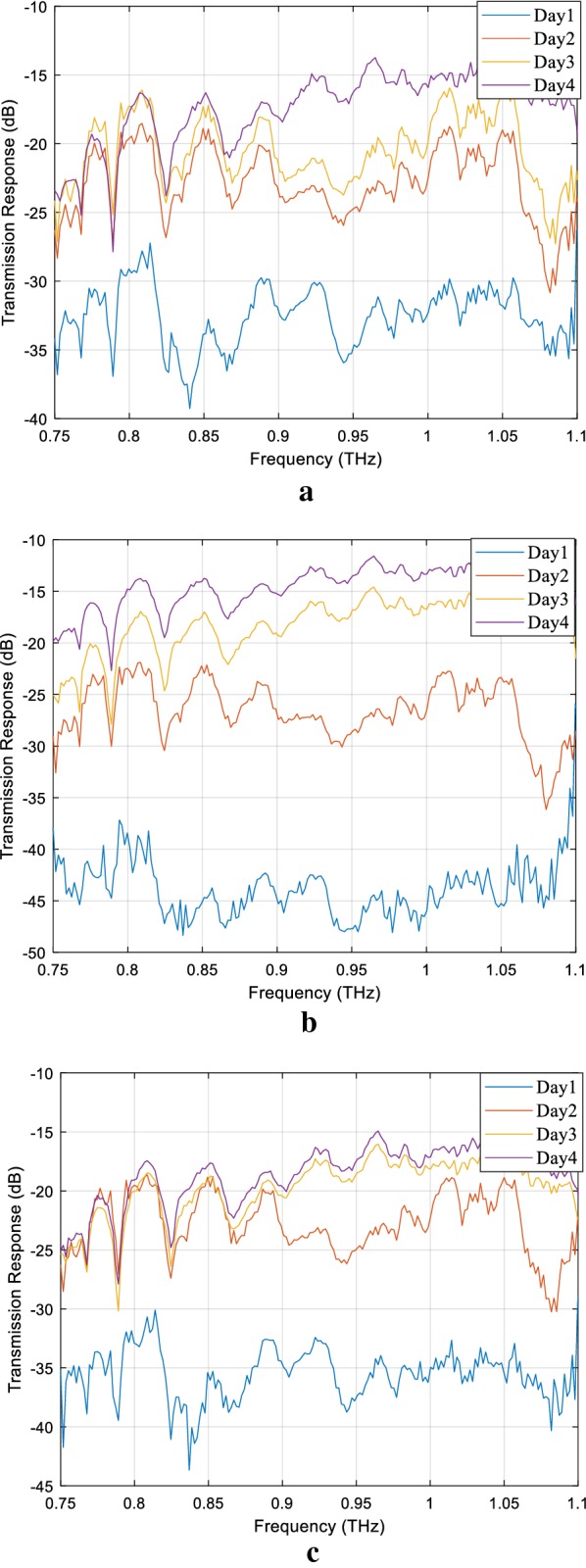
Transmission response of coffee, pea-shoot, and spinach leaves observed on four different days in the frequency range of 0.75 to 1.1 THz. **a** Coffee. **b** Pea-shoot. **c** Baby spinach

We used Matlab R2019a for preprcoessing of the data as well as classification in the form of supervised learning. The measurements data for all three fresh plant leaves were obtained in the Radio Frequency Laboratory at the University of Glasgow for four consecutive days. For each observation, all distinct leaves were placed between the two waveguides, and observations were recorded. Both the transmission coefficients (S_12_, S_21_) and reflection (S_11_, S_22_) were determined from the measurements. The overall experimental setup for measuring the WC of all fresh plants’ leaves is shown in Fig. 1. In this work, the focus was mainly to consider the transmission response as features for all three leaves and is shown in Fig. 2. Every day, the duration of measuring the THz transmission response was approximately 9–10 h to observe various degree of WC in all three leaves was, and measurements were recorded after every 120 min. This process was repeated for four consecutive days. Hence, the total number of observations collected for coffee, pea-shoot and baby-spinach for continuous 4 days are listed in Table 1. Table 1 shows the difference in the number of observations of leaves which indicates that each leaf had a variable degradation in WC during the 4 days of measurements. On each day, 10 rounds of weight measurements were recorded over the span of 4 days and converted into WC using (1) [17, 21, 24].

**Table 1:** Observations collected for three leaves for four consecutive days

Leaves	Number of observations
*Coffea arabica*	127
Pea shoot	76
Baby spinach	54

1 $$WC = \frac{{W_{time} - W_{dry} }}{{W_{fresh} }} \, \times \, 100\%$$

Upon close analysis of Fig. 2, it was depicted that coffee, pea-shoot and baby-spinach leaves exhibited distinct responses on all 4 days. On day 1, the transmission response for all leaves was significantly low due to the presence of high volumetric WC in leaves. Notably, pea-shoot revealed a response in the range of − 40 dB to − 45 dB reflecting a distinct characteristic from other leaves. The difference in transmission response also highlighted a physiological process, affecting the variability of the water dynamics in these leaves.

### Feature extraction methods

**Fig. 3:**
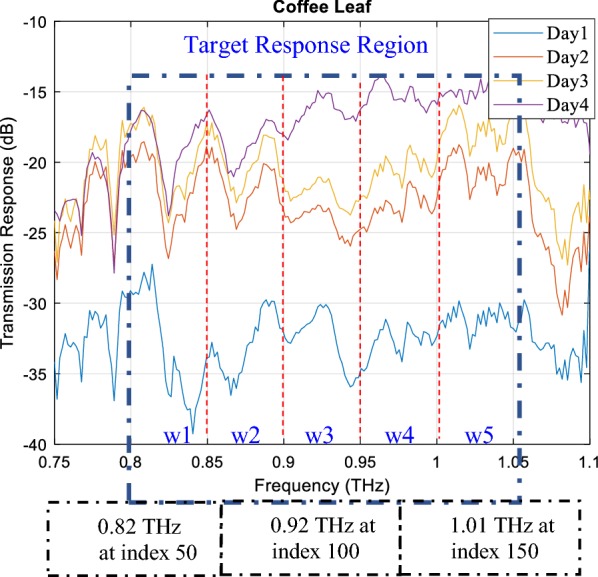
Identification of target response region (TRR) to consider only relevant and important features for the feature extraction process

During the THz experimental campaign of measuring the transmission response of leaves, the observations spawned by Swiss*to*12 (MCK) were erratic (exhibiting unwanted excessive variations), especially at both ends of frequency range from 0.75 to 0.80 THz and 1.05 to 1.1 THz as shown in Fig. 2 [25]. The effect of this undesired noise could be crucial and may have produced false observations about the WC in leaves in rest of the frequency region. Inevitably, it would have produced counterfeit classification results by classifiers about the quantification of WC in leaves. Furthermore, any erroneous estimation of WC in leaves would ultimately affect their overall biological and physiological process of growth. Hence, it was significant to discover the sensitive frequency region (SFR) with the minimum effects of any unwanted errors in the overall observation data. Therefore, the target response region (TRR) was established where observations could be visibly distinguished without any overlap for leaves on all different days. The TRR for coffee leaf was selected in the range of 0.82 to 1.05 THz, as shown in Fig. 3. Furthermore, useful observations would also have a fruitful impact on overall classification outcome.

**Fig. 4:**
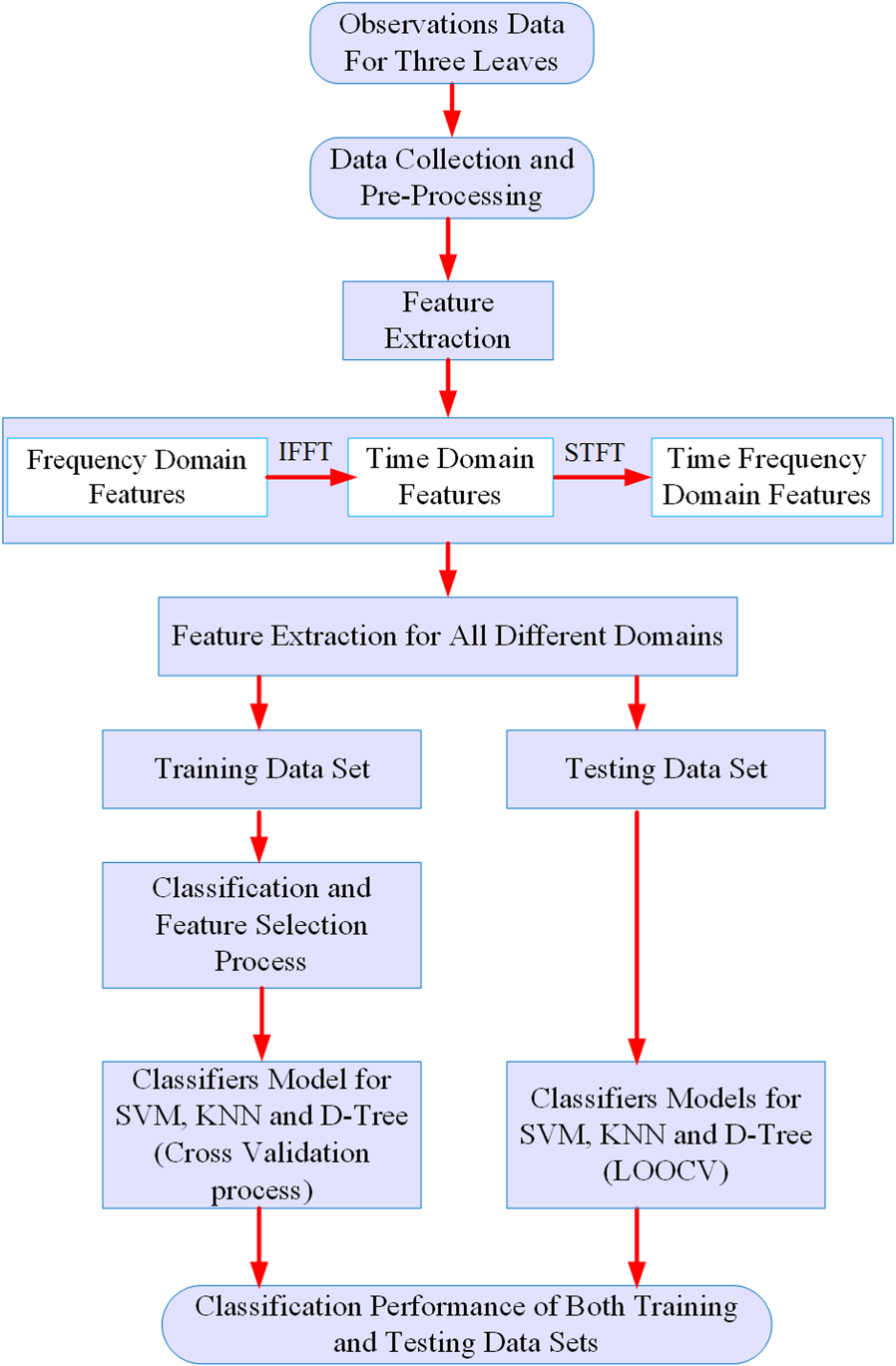
The flowchart of the proposed algorithm implementation process

Researchers have suggested and applied many features extraction techniques to execute the classification accuracy [26]. In this work, observations recorded were in the frequency domain had to be converted into time and time–frequency domain to further minutely observe the behaviour of WC in various leaves by analysing statistical features. Hybrid combinations of multi-dimension features domain would have a favourable response in classification accuracy by reducing overall dimensions of initial features [26]. The frequency-domain was converted into the time domain and time–frequency domain by applying Inverse Fast Fourier Transform (IFFT) and Short-Time Fourier Transform (STFT) respectively [26]. The list of different domains is summarised in Table 2. Hence, out of 201 features, only 25 significant features were considered which comprised of 11, 10, and 5 in the time-domain, frequency domain, and time–frequency domain respectively as indicated in Table 2. The block diagram of the proposed classification system for different days based on multi-domain features extraction approach is shown in Fig. 4.

**Table 2:** Feature extraction technique for all three leaves

Time domain (statistical features)	Serial no.	Frequency domain features	Serial no.	Time–frequency domain	Serial no.
*No. of features*	*11*	*No. of features*	*10*	*No. of features*	(*4*)
Mean	1	CPSD (D = 20)	12	Subband1	22
Variance	2	CPSD (D = 40)	13	Subband2	23
(MAD)	3	CPSD (D = 60)	14	Subband3	24
Skewness	4	CPSD (D = 80)	15	Subband4	25
Kurtosis	5	CPSD(D = 100)	16		
Standard deviation	6	PSD (D = 20)	17		
MAV	7	PSD (D = 40)	18		
75th (Q_3_)	8	PSD (D = 60)	19		
25th (Q_1_)	9	PSD (D = 80)	20		
PCC	10	PSD (D = 100)	21		
IQR	11

### Evaluation of frequency features extraction

Since the data obtained from VNA was in the frequency domain, it was significant to focus mainly on the region that gives the maximum and the accurate information about the existence of WC in all three leaves. For this purpose, as mentioned earlier, TRR was mainly required. In this regard, five windows bins with a width of 20 were initiated in the middle region (0.92 THz at index = 100) and symmetrically expanded to both sides of the frequency region. From Fig. 3, the data under the observation of the selected area can be seen, and was applied to the rest of two leaves as well. In addition, the frequency domain features included a cross-power spectral density and variance of power spectral density and is given by the Eqs. (2) and (3) [27] respectively. From the Eq. (2), $Y_{l}^{n} (a)$ represents the transmission response of the reference signal. In Eq. (3), $T(a)$ implies the transmission response of *l*-th leaf on an nth day. Here, *‘w’* is considered as the width of the frequency window as depicted in Fig. 3.

2 $$Var\{ Y_{ll} (a)\} = \frac{1}{w}E \, [\{ Y_{l}^{n} (a)*.Y_{l}^{n} (a)\} ]$$

3 $$\text{max} \{ Y_{lm} (a)\} = \text{max} \left(\frac{1}{w}E\{ (T(a)*.Y_{l}^{n} (a))\} \right)$$

### Evaluation of time features extraction

For statistical features, the transmission response of time-series of THz pulse was observed from days 1 to 4, indicating any possibilities of WC in leaves. Therefore, it was required to convert frequency domain data into the time-domain features to observe meaningful THz pulse. For this purpose, 11 time domain features were employed and they are mean, median, mean of absolute value (MAV), standard deviation (STD), mean of absolute deviation (MAD), skewness and kurtosis, Pearson correlation coefficient (PCC) [28], 25th percentile (Q1), 75th percentile (Q3), and Interquartile Range (IQR) [29]. In which, mean and standard deviation were particularly useful to provide significant information about the distribution of data [25]. Skewness produced meaningful information about the irregularities of the examined area and its distribution around its mean [29, 30]. Moreover, kurtosis presented a measure of evenness relative to a standard distribution [29]. Q3 and Q1 showed how the observation data were dispersed in the two sides of the median. PCC was used to measure the linear relationship between the time-domain waveforms of the sample and the reference signal [29]. IQR was also used to measure the variability of the dataset and shows the difference between Q3 and Q1 while measuring the data distribution set. This information was also helpful in terms of excluding irrelevant data [29].

### Evaluation of time–frequency features extraction

The demand for considering time–frequency technique such as Short-Time-Fourier-Transform (STFT) and Wavelet Transform (WT) was mainly to obtain the detailed information of THz pulses in this domain [31] The WT technique was more appropriate to analyse short-term THz pulse produced because of any diminutive variations occurred at the cellular level, reflecting an information of WC in leaves. After the de-noising process, the wavelet spectrum features were extracted by considering the power of various sub-bands at different levels as defined in Eq. (4) to extract the time-domain features [32, 33].

4 $$E(j,i) = \frac{1}{N}\sum\limits_{k = 1}^{N} {[P_{k} (j,i)]^{2} }$$

In the above equation, j denotes the level of wavelet decomposition and ith indicates as the sub-band and ‘N’ is the number of wavelet coefficients. $P_{k} (j,i)$ is basically the wavelet coefficient vector of ith sub-band in the jth level. Hence, $E(j,i)$ denotes the average power value of ith sub-band at the jth level. Table 2 summarised the features extracted from time, frequency, and time–frequency domains. Each feature is assigned one serial number from 1 to 25, in which, 1-11, 12-11 and 22-25, were the serial numbers of time-domain, frequency-domain, and time–frequency domain features, respectively.

### Proposed classification algorithm and parameters selection

In this section, the significant of optimum parameters were determined for three classifiers including SVM, KNN, and D-Tree. In addition, on the basis of suitable parameters selection, classification algorithm was developed, and its performance was evaluated for precise estimation of WC in leaves.

### Selection of optimal parameters values

In order to develop an algorithm for three classifiers various parameters were considered. For accurate classification results, it was significant to have optimal parameters for classifiers. Here, three classifiers which include SVM, KNN and D-Tree were considered for precise estimation of WC in three leaves from day 1 to 4. For each classifier, a series of values for tuning the process with optimal parameters were determined to achieve the highest overall classification accuracy and performance of classifiers were also analysed. For SVM, two parameters i.e. the optimum parameters of cost (C) and kernel width parameter (ϒ) are required to be set when applying the SVM classifier with radial basis function (RBF) kernel to achieve the optimized SVM algorithm [34]. The ‘C’ parameters helped to decide the actual size of misclassification permitted for non-separable training data and adjusted the rigidity of the training data [35]. Larger values might lead to an over-fitting model and vice versa. The kernel width parameter (ϒ) facilitated the shape of the class-dividing hyperplane, and increasing or decreasing the value of (ϒ) could influence the shape of the class-dividing hyperplane, and it eventually disturbed the classification accuracy. For this purpose, a series of values were assessed and to establish the most suitable value for ‘C’ for available data, and finally “1” was chosen for ‘C’, and “0.38” was selected for (ϒ).

The basic theory behind the KNN was to discover a group of ‘k’ samples that appeared to be nearest to the unknown samples [34]. From k-samples, the label of unknown samples could be determined by evaluating the average values for class-attributes [34, 35]. Thus, tuning this fundamental parameter of k-sample played a significant role in achieving the ultimate performance of this classifier. For this purpose, a different range of values was established, and finally, it was settled in the range from 1 to 5 to recognize the optimal ‘k-value’ for all training sample sets. For D-tree, again the various range of numbers for splits in D-test was analysed for the available data to identify the optimum parameter. Eventually, it was set to 5, and the rest of the settings were retained as default values for this classifier.

## Results

### Classification accuracy and features selection

In this study, the performance of proposed classifiers including SVM, KNN, and D-Tree was assessed on raw data and on individual domain features. Furthermore, all classifiers showed distinct performances on individual domain features. Henceforward, classification accuracy for a hybrid combination of all three domains was also obtained. Towards the end, features selection was illustrated using the various state-of-the-art techniques.

### Assessment of classifiers on raw data

Before processing the classification accuracy of raw data, the frequency range of 0.75 to 1.1 THz was considered for executing classifications. Also, all observations were taken as separate features and performance of the classifiers were tested on all features. The main aim here was to evaluate the classifier response by examining all observations of three leaves at different days at every frequency point. Hence, three classifiers, including SVM, KNN, and D-tree performances were tested to estimate the WC in leaves more accurately and precisely. The classifiers were trained and validated using a k-fold and feature set was partitioned into 10 “folds” randomly. The observations data was partitioned into 70% and 30% training and testing data, respectively. Table 3 listed the average classification accuracy results of all three classifiers.

**Table 3:** Raw data classification results for three leaves

Accuracy (%)	Coffee	Peashoot	Baby spinach
SVM	80.22	76.26	75.78
KNN	75.1	72.95	74.98
DTree	76.24	69.58	76.93

**Fig. 5:**
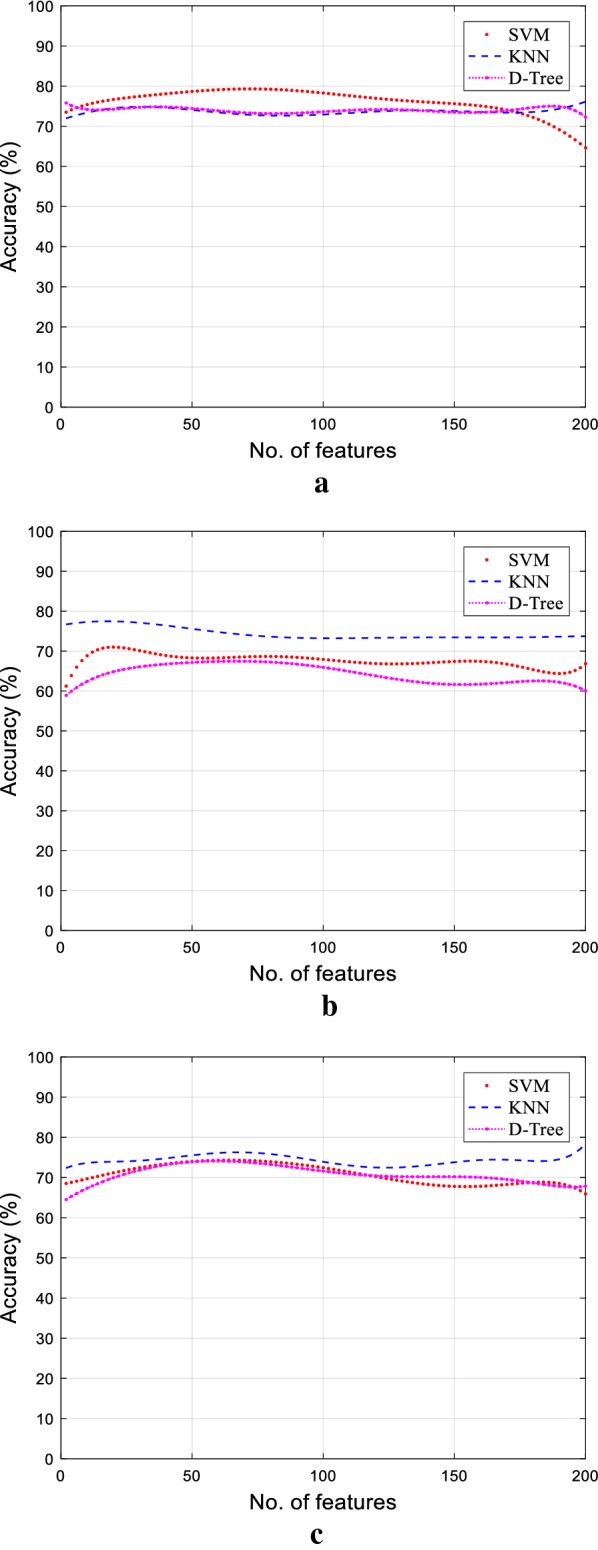
Classification performance of raw data for coffee, pea shoots and spinach leaves considering all features from 0.75 to 1.1 THz. **a** Coffee. **b** Pea shoot. **c** Baby spinach

By close investigations of results in Fig. 5 and Table 3, it was depicted that classification accuracy for all leaves found in the range of 70–75%. This low accuracy reflected some redundant or irrelevant features in the overall 201 features points, which badly affected the classification accuracy. Therefore, the performance of all three proposed classifiers could be improved by reducing undesired features and selecting more meaningful and informative features to produce an accurate estimation of WC in all three leaves. Thus, the purpose of observing the performance of the classifiers on raw data was mainly to explore the TRR, as explained in the previous section.

### Assessment of classifiers for individual and hybrid combination features

Once the parameters were set for all classifiers, its performance was investigated on different domain features individually and a hybrid combination of all three domain features. So, its performance accuracy was accomplished, and Tables 4, 5 and 6 demonstrated the classification accuracy results for coffee, pea-shoot and baby spinach, respectively. The classification accuracy results were obtained for 25 extracted features. These 25 features were comprised of time domain, frequency domain and time–frequency domain features. Upon close analysis, the classifiers performed relatively better for coffee leaf compared to pea shoot and baby spinach for set parameters, which were selected before the classifier model was produced.

**Table 4:** Classification results for coffee leaf

Classification accuracy (%)	Time domain features (11), %	Frequency domain features (10), %	Time–frequency domain features (4), %
SVM	92.6	93.0	91.6
KNN	90.0	91.8	89.4
Decision tree	91.2	90.7	91.2

**Table 5:** Classification results for pea shoot leaf

Classification accuracy (%)	Time domain features (11), %	Frequency domain features (10), %	Time–frequency domain features (4), %
SVM	86.6	79.2	80.6
KNN	79.0	78.8	81.4
Decision tree	81.2	81.7	82.2

**Table 6:** Classification results for baby spinach leaf

Classification accuracy (%)	Time domain features (11), %	Frequency domain features (10), %	Time–frequency domain features (4), %
SVM	82.6	81.1	84.6
KNN	81.0	78.8	81.4
Decision Tree	78.2	79.7	82.2

Moreover, it also showed that the precise estimation of WC presence in coffee leaf from day 1 to day 4 had been substantially improved compared to other leaves. Since the content of water is vital indicator for explaining the plants overall vitality and growth processes, therefore, timely detection of any deficiency in WC plays a signification role in monitoring the health status of leaves effectively. After the individual performance of three features domain, another attempt was made to assess the performance of the classifier for hybrid combinations of all three domain features collectively. Table 7 displayed the classification accuracy of all three classifiers for all three leaves. In this condition, classifiers were trained and cross-validated by applying k = tenfolds, and the performance of all three classifiers was obtained. These classifiers, including SVM with RBF kernel, KNN with k = 5 and D-Tree, were trained and cross-validated by applying k = tenfolds. The observations data was partitioned into 70% and 30% training and testing data, respectively. By comparing the results of hybrid combinations with individual classification performance, it was discovered that the combination of features produced an improvement in classification accuracy for all three leaves. Previously, individual classification only enhanced the coffee leaf, whereas the combination of all three domain collectively improved the performance for other leaves, including pea shoot and baby spinach.

**Table 7:** Classification results of hybrid combination features for all leaves

Classification accuracy of three leaves	SVM, %	KNN, %	D-Tree, %
Coffee	94.46	93.76	91.15
Pea shoot	93.42	91.62	90.64
Baby spinach	91.13	90.38	89.01

### Optimization and feature selection

In this work, the aim was to remove any redundant or irrelevant features through the feature selection technique to enhance the classification performance by lessening the computational cost for deployment. The methods for feature selection contain filtering methods which were based on the evaluation of the relevance of features, and other wrapper methods were based on a strong search of a different set of features [36]. We considered three feature selection algorithms named as sequential forward selection (SFS), sequential backward selection (SBS) and Relief based selection algorithm (Relief-F) to execute the feature selection process [37]. Out of these three algorithms, SFS and SBS were considered the two most empirical selection algorithms [37]. SFS begins with an empty set and integrates the most suitable feature in every step, and exhibiting a high accuracy by employing a classifier until the pre-defined features are tallied up [37].

**Fig. 6:**
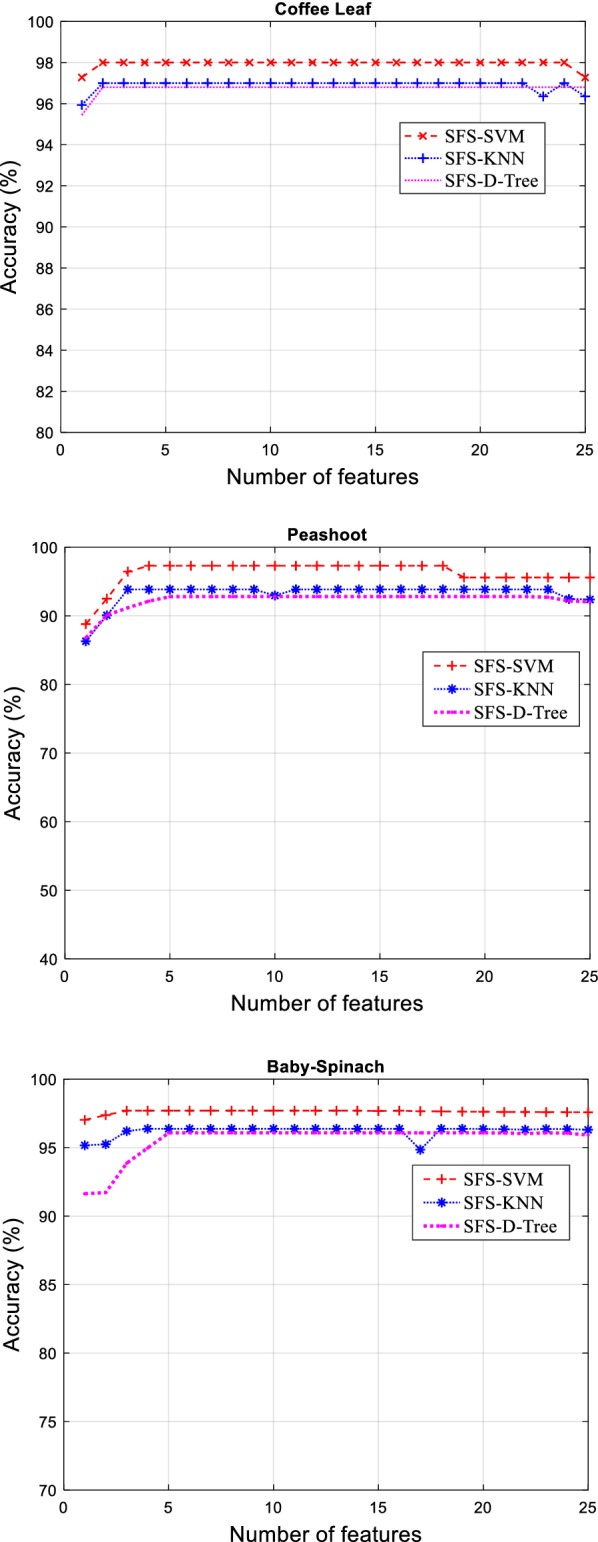
Classification performance of classifiers using feature selection technique SFS for coffee, pea shoot and baby spinach leaves

On the contrary, SBS operates opposite to the SFS and begins with full occupied features and disposed of unmatched features in every step by specific criterion function till the pre-defined features are permitted [38]. Intriguingly, Relief-F can propose a more efficient technique compared to SFS and SBS and comprehend the relations of features to compute the weights of the features for accurate ranking and selection irrespective of any dependency on specific classifiers [39]. Figure 6 depicted the performance of SFS features selection for coffee, pea-shoot and baby spinach leaves using three classifiers.

From Fig. 6, it was noticed that SVM performed considerably better for all leaves compared to other classifiers using different selection techniques. In addition, Tables 8, 9, and 10 displayed the classification accuracies for coffee, pea shoot and baby spinach leaves, respectively, using various features selection techniques with the required number of features. By applying a features selection algorithm to classifiers, they produced an improvement of 4%, 3% and 6% for coffee, pea-shoot, and baby spinach leaves using SVM classifiers through SFS technique. The performance of KNN for coffee, pea-shoot, and baby spinach leaves also presented progress in results by 3%, 4%, and 5% correspondingly. These tables indicated the different combinations of features including frequency, time-domain, and time–frequency domain features for classification accuracy.

**Table 8:** Classification performance for coffee leaf by applying tenfold validation using proposed algorithm with selected features

Feature selection methods	Classifiers	Serial num. of features	Total no of features	Accuracy (%)
SFS	SVM	24	1–19, 21–25	98.5
	KNN	22	1–6, 8–11, 13–21, 23–25	97.2
	D-Tree	24	1–23, 24	96.5
SBS	SVM	24	1–19, 21–25	98.6
	KNN	24	1–21, 23–25	97.6
	D-Tree	24	1–23, 25	96.2
Relief-F	SVM	10	2, 4, 10, 11, 17–21, 25	97.1
	KNN			95.9
	D-Tree			96.8

**Table 9:** Classification performance for pea shoot leaf by applying tenfold validation using proposed algorithm with selected features

Feature selection methods	Classifiers	Serial num. of features	No of selected features	Accuracy (%)
SFS	SVM	18	1–6, 8–14, 17, 19, 20, 22, 25	97.2
	KNN	13	1–5, 9–11, 18–20, 23, 25	94.4
	D-Tree	7	2, 4, 5, 6, 17, 18, 19	93.1
SBS	SVM	3	13, 19, 22	96.8
	KNN	5	7, 12, 17, 19, 20	94.9
	D-Tree	2	8, 20	92.3
Relief-F	SVM	12	2, 4, 10, 11, 17–21, 23–25	98.6
	KNN			99.1
	D-Tree			95.5

**Table 10:** Classification performance for baby spinach by applying tenfold validation using proposed algorithm with selected features

Feature selection methods	Classifiers	Serial num. of features	Total no of features	Accuracy (%)
SFS	SVM	24	1–12, 14–25	97.9
	KNN	23	1–14, 17–25	96.4
	D-Tree	5	3, 5, 17, 20, 21	96.1
SBS	SVM	23	1–11, 13, 15–25	96.8
	KNN	24	1–13, 15–25	94.5
	D-Tree	5	7, 8, 9, 11, 15	93.2
Relief-F	SVM	17	2, 4–7, 10, 11, 15–21, 23–25	98.6
	KNN			99.1
	D-Tree			95.5

As explained in the previous section, it was aimed at reducing the computational time using feature selection techniques. So, in this study, Table 11 presented the overall execution time taken by three classifiers for generating results using various feature selection techniques. It was established that execution time taken by classifiers for selected features by performing tenfold, cross-validation showed considerable enhancement compared to extract features. For example, coffee leaf exhibited an improvement of 15%, 11.9% and 6.5% in computation time for SVM, KNN and D-Tree, respectively, using SFS technique. For pea-shoot, an upgrade of 21.28%, 10.01%, and 8.53% was noticed in operating time for SVM, KNN and D-Tree classifiers, respectively. Lastly, in baby spinach leaf, considering SFS technique, SVM showed an upgrade of 21.28% in SVM, 10.01% in KNN, and 8.53% in D-Tree operating times. These outcomes indicated that selecting the most relevant and vital features not only enhanced the overall operation time for classifiers but also improved the classification as confirmed with Tables 8, 9 and 10. Hence, Table 11 is significant for finding the performance of classifiers with less computation time for execution of classification accuracy. In this work, the core purpose was not only to achieve less computation time but also to select relevant features with maximum information using various feature selection techniques. In addition, it could utilize less time and produce maximum accuracy for estimation of WC in plants leaves to maintain a healthy physiological status.

**Table 11:** Classification performance of all classifiers by applying tenfold validation using proposed algorithms with selected features

Feature types and feature selection methods	Computation time (s)		
	SVM	KNN	Decision tree
Coffee leaf			
Extracted features	0.7282	0.5309	0.4021
Selected features			
SFS	0.5706	0.4123	0.3371
SBS	0.6456	0.4240	0.3202
Relief-F	0.6252	0.4842	0.3582
Baby spinach leaf			
Extracted features	0.8975	0.4265	0.4053
Selected features			
SFS	0.6062	0.4128	0.1071
SBS	0.4259	0.3576	0.3247
Relief-F	0.4485	0.3875	0.3490
Peashoot leaf			
Extracted features	0.6825	0.4405	0.4196
Selected features			
SFS	0.4699	0.3404	0.3343
SBS	0.6504	0.1734	0.3149
Relief-F	0.5088	0.3766	0.3759

## Discussion

In this section, the performance of three proposed classifiers were assessed by employing two commonly quality metrics such as sensitivity or recall (also known as true-positive rate) and specificity (also called false-positive rate) [29, 40]. Here, sensitivity values indicated the possibility of correct identification of labelled class from the remaining target classes [29]. In contrast, specificity showed the probability of appropriate classification as non-target classes from the remaining un-aimed classes [40]. The purpose of utilizing these two widely accepted metrices [29, 40] was mainly to detect any misclassification that could occur, leading to inaccurate information about WC in leaves for four consecutive days.

These two-quality metrices depicted the performance of classifiers ranging values from 0 to 1 on days 1 to 4, indicating the presence of WC in all three leaves. Table 12 illustrated the performance of all classifiers using a feature selection method and showed the WC presence in all three leaves from day 1 to 4. From Table 12, it was also perceived that SVM outperformed other classifiers for a coffee leaf on different days. Moreover, the assessment of quality metrics for a coffee leaf on days 1 and 4 performed noticeably better, revealing the freshness and staleness of leaf. These results also discovered that the presence of WC on day 1 was high and low on day 4, which helped the classifier to execute the improved performance. Furthermore, it was worth noting that the classification accuracy for all leaves on days 2 and 3 was slightly challenging when the presence of WC in leaves was found in the range of 20% to 50% approximately.

**Table 12:** Classification performance of all classifiers by applying leave-one-observation-cross-validation techniques with selected features

Quality metrics	Water content (%)	SVM	KNN	D-Tree
Coffee leaf				
Day 1	82.84			
SENS		1	1	1
SPEC		1	1	1
Day 2	41.22			
SENS		1	0.929	0.976
SPEC		0.988	0.965	1
Day 3	12.34			
SENS		0.963	0.889	1
SPEC		1	0.912	0.99
Day 4	0.51			
SENS		1	1	1
SPEC		1	1	1
Peashoot				
Day 1	76.84			
SENS		1	1	1
SPEC		1	1	1
Day 2	49.22			
SENS		1	0.892	1
SPEC		0.962	0.982	0.971
Day 3	18.91			
SENS		0.545	0.727	0.636
SPEC		0.984	0.967	0.984
Day 4	0.21			
SENS		0.919	0.85	0.833
SPEC		0.987	0.85	0.933
Spinach				
Day1	71.14			
SENS		0.995	1	1
SPEC		1	1	1
Day2	34.22			
SENS		1	1	1
SPEC		0.976	1	1
Day3	10.34			
SENS		0.909	0.545	0.851
SPEC		0.923	0.949	0.897
Day4	0.10			
SENS		0.727	0.818	0.636
SPEC		0.974	0.872	0.949

Considering the real-life scenario, the proposed methodology can be substantial by observing the performance of the classifiers for leave-one-observation-out cross-validation method to achieve different days classifications accuracy and for accurate estimation of WC in leaves. This proposed method evaluated the actual performance of the classifier model by randomly selecting each observation from the dataset considered as a validation set, while the remaining observations were taken as the training set. This process continued until all observations from the dataset were nominated for the validation set for at least one attempt. Table 12 illustrated the accuracy of the classifications of all leaves for each day by applying the leave-one-observation-out cross-validation technique.

From Table 13, it was perceived that SVM classification accuracy outperformed other classifiers for all leaves by showing minimum variance. It also displayed that variability in WC of leaves over the course of four consecutive days. Furthermore, it was also noticed that for both days 1 and 4, classifiers produced maximum accuracy reflecting a high and low WC on days 1 and 4, respectively. Whereas on days 2 and 3, SVM performance stayed in the range from 92.6 to 100%, KNN yielded a range of 78.4 to 100%, and D-Tree produced a range of 74.2 to 100%. Hence, it was concluded that SVM achieved a better classification accuracy range on days 2 and 3 compared to other classifiers. Thus, the aim of applying leave-one-observation-out cross-validation technique was to evaluate the consistency of classifiers by assessing all observations of different samples on different days as depicted in Table 13. It was also strongly aimed to assess the performance of the proposed ML algorithm with the incorporation of THz for real-time applications in monitoring any diminutive variations of WC in plants leaves to help in developing digital agricultural systems.

**Table 13:** The confusion accuracy with leave-one-observations-out cross-validation method of all leaves for each day along with monitoring the water content values for each day

Samples	Classes	Classifiers test accuracy performance (%)			Water content (%)
		SVM	KNN	D-Tree	
Coffee leaf	Day1	100	100	100	82.84
	Day2	95.2	88.1	100	41.22
	Day3	100	92.6	92.3	12.34
	Day4	100	100	100	0.71
	Variance	0.58	1.09	0.92	
Peashoot leaf	Day1	100	100	100	76.84
	Day2	100	87.5	87.5	49.22
	Day3	93.6	78.4	74.2	18.91
	Day4	95.0	89.3	91.7	0.21
	Variance	1.55	2.27	3.60	
Baby spinach leaf	Day1	100	100	100	71.14
	Day2	100	100	100	34.22
	Day3	92.6	88.6	75.5	10.34
	Day4	94.7	89.7	91.3	0.10
	Variance	1.76	2.90	4.60	

## Conclusions

In this paper, a novel machine learning (ML) driven approach was proposed to accurately determine the health status of plants leaves terahertz (THz) waves. In this process, transmission response of leaves was measured for four consecutive days, where each of the 201 frequency points were used as a feature. We performed feature selection to discard any irrelevant and spurious features that could give false observations about the water content (WC) in leaves. In this study, results showed that the performance of classifiers was drastically improved by identifying more relevant and important features that could can yield maximum information about WC in leaves, to maintain healthy physiological status of leaves. The selection of useful features also reduced the computation time for the execution of classifications by all three classifiers, which was also one of an ultimate objective. Moreover, the comprehensive cross-validation methodology demonstrated that, in most cases, support vector machine SVM yielded highest classification accuracy compared to other classifiers. It was observed that SVM achieved relatively more reliable results for predicting the accurate WC estimation in three leaves for four consecutive days.

This paper demonstrates the potential and establishes a notable integration of machine learning (ML) using terahertz (THz) waves to assess the real-time information of WC in various plants’ leaves. In an era, where most of the farmlands around the globe are water-stressed, the outcomes of this study can help in the design and implementation of smart, sustainable digital agricultural technologies, which is of high importance to boost the overall crops productivity.
